# A Randomized, Double-Blind, Placebo-Controlled Trial of a Polyphenol Botanical Blend on Sleep and Daytime Functioning

**DOI:** 10.3390/ijerph18063044

**Published:** 2021-03-16

**Authors:** Andrew S. Tubbs, Kathryn E. R. Kennedy, Pamela Alfonso-Miller, Chloe C. A. Wills, Michael A. Grandner

**Affiliations:** 1 Sleep and Health Research Program, Department of Psychiatry, University of Arizona College of Medicine—Tucson, Tucson, AZ 85724, USA; katkennedy@email.arizona.edu (K.E.R.K.); chloecawills@psychiatry.arizona.edu (C.C.A.W.); grandner@email.arizona.edu (M.A.G.); 2 Department of Psychology, Northumbria University, Newcastle-upon-Tyne NE1 8ST, UK; pam.alfonso-miller@northumbria.ac.uk

**Keywords:** nutrition, sleep quality, polyphenol, cognition, dietary supplement

## Abstract

Despite the high prevalence of subclinical sleep disturbances, existing treatments are either potent prescription medications or over-the-counter supplements with minimal scientific support and numerous side effects. However, preliminary evidence shows that polyphenols such as rosmarinic acid and epigallocatechin gallate can support healthy sleep without significant side effects. Therefore, the present study examined whether a polyphenol botanical blend (PBB) could improve sleep and/or daytime functioning in individuals with subclinical sleep disturbances. A total of 89 individuals completed a double-blind, randomized trial of daily treatment with PBB (*n* = 43) or placebo (*n* = 46) 30 min before bed for 30 days. Participants were monitored for changes in sleep (by sleep diary and an activity tracker), mood, and neurocognitive functioning. After 30 days, PBB improved diary sleep quality (*p* = 0.008) and reduced insomnia severity (*p* = 0.044) when compared to placebo. No other changes in sleep outcomes were observed. Additionally, PBB did not impair neurocognitive functioning, and some improvement was noted in vigilant attention, working memory, and risk assessment. Among individuals with subclinical sleep disturbances, PBB improved sleep quality, insomnia severity, and neurocognitive functioning over placebo. These findings indicate that polyphenol compounds may be useful for improving certain aspects of sleep without compromising neurocognitive functioning.

## 1. Introduction

Sleep disturbances are associated with impaired memory, cognitive dysfunction, and emotional dysregulation [1,2,3]. For example, insomnia disorder is a common cause of disrupted sleep and affects as many as 10 to 20% of the U.S. population [4]. However, nearly half of adults experience transient insomnia [5], indicating that broad swathes of the population are vulnerable to sleep disturbances that degrade physical and mental health.

Unfortunately, few interventions exist for individuals with subclinical sleep disturbances. While cognitive behavioral therapy for insomnia is the recommended first-line treatment for insomnia due to its efficacy and minimal side effects [6,7], there are not enough trained therapists to treat individuals with chronic insomnia, let alone subclinical sleep problems [8]. Consequently, many patients seek prescription antidepressants and hypnotics, which are heavily sedating, less effective, have more side effects, and can result in long-term dependence, or over-the-counter sleep aids such as diphenhydramine or doxylamine, which can result in cognitive impairments and daytime sleepiness [9,10,11]. Herbal supplements are also commonly used, such as valerian root (*Valeriana officinalis*), lemon balm (*Melissa officinalis*), and chamomile (*Matricaria recutita* and *Chamaemelum nobile*). However, the evidence for such substances is usually derived from small, uncontrolled studies lacking valid measures of sleep [12,13], and complaints of next-day grogginess, dizziness, headaches, and nausea are common [14].

By contrast, emerging evidence indicates that polyphenols, such as rosmarinic acid (RA) and epigallocatechin gallate (EGCG), may be effective sleep aids [15,16,17,18]. RA has potent antioxidant effects in the brain, thus providing neuroprotective benefits, and may affect sleep by modulating GABA and acetylcholine [16,17,19,20,21,22], while EGCG can attenuate corticosterone release to downregulate the hypothalamic–pituitary–adrenal axis to provide anxiolytic and hypnotic effects [15,23]. Dietary intake of polyphenols, including RA and EGCG, is associated with improved sleep quality in healthy adults [24], as well as reduced anxiogenic behavior and increased sleep time [25]. Thus, polyphenol compounds may be effective at improving sleep without the negative side effects of other pharmacological treatments.

Therefore, the present randomized placebo-controlled trial evaluated a polyphenol botanical blend (PBB) as a sleep aid in individuals with minor sleep disturbances. The primary hypotheses were that PBB supplementation would improve sleep onset latency, as well as percentage of time in rapid-eye movement (REM) sleep due to hypothesized cholinergic effects. The secondary hypotheses were that the use of PBB would improve other aspects of sleep, such as sleep efficiency, sleep quality, or insomnia symptoms. Additional analyses explored possible improvements in mood and neurocognitive functioning.

## 2. Materials and Methods

### 2.1. Overview

A 30-day double-blind, randomized, 1-to-1 placebo-controlled trial was conducted to compare the effects of daily PBB supplementation on sleep and daytime functioning. Subjects were assessed at 0-, 7-, and 30-days post randomization. Sleep was monitored by sleep diaries and commercial activity trackers throughout the trial. This study was approved by the University of Arizona Institutional Review Board, conducted in accordance with the Declaration of Helsinki, and registered with clinicaltrials.gov (NCT03567343). All subjects provided informed consent prior to randomization.

### 2.2. Participants

Subject recruitment occurred by self-referral, social media, and flyer advertising in Tucson, Arizona, USA. Participants were aged 22–50, in general good health, had a body mass index between 18.5 and 29.9 kg/m^2^, and had not used nicotine in the past 6 months. A score of 3 or higher on the Pittsburgh Sleep Quality Index was required for participation.

Participants were excluded if they had an active infection, uncontrolled hypertension, a major psychiatric disorder as determined by the Mini International Neuropsychiatric Inventory [26], a history of cancer within 5 years, a history of unconventional sleep pattern, a diagnosed sleep disorder, or a chronic medical condition that could affect energy/fatigue levels. Participants were also excluded if they were currently experiencing a major depressive episode as determined by current Patient Health Questionnaire–9 [27] score, were allergic to study products, consumed more than 400 mg of caffeine per day in the past 2 weeks, had used any psychotropic medications, stimulants, cannabis, non-registered drug products, or illicit substances in the past 4 weeks, were at risk of drug or alcohol abuse, or had used any sleep aids in the past 2 weeks. Finally, women who were pregnant, planning to be pregnant, lactating, or unwilling to use a medically approved form of contraception were excluded.

### 2.3. Procedures

This study took place between May 2017 and September 2018. A final study sample of *N* = 100 (50 per group) was calculated based on proprietary pilot data conducted by the study sponsor. PBB and placebo were packaged into identical capsules and bottles, masked, and sent to the investigative site. The PBB is a 485 mg dose containing at least 120 mg polyphenols (and at least 65 mg rosmarinic acid and epigallocatechin gallate), and no more than 4.85 mg of caffeine (≤1%) per dose. Once participants were screened as eligible and provided informed consent, they were randomized to either PBB or placebo using random number assignment (rand function in Excel) and sequentially numbered bottles. Study staff involved in enrollment, data collection, and analysis were not involved in generating the randomization sequence and were unaware of participants’ group status until after the trial was complete. Similarly, participants were blinded to their treatment status. Once randomized, participants were provided with an activity tracker (Fitbit Charge 2, Fitbit, San Francisco, CA, USA) and directed to complete a daily sleep diary. Participants were instructed to take the supplement 30 min before bedtime starting on Day 3 (to allow for pre-treatment baseline data collection), and to maintain a consistent diet throughout the study period. Additionally, alcohol consumption was limited to ≤14 drinks per week, no more than 4 drinks at a time, and no more than 1 drink within 4 h of bedtime. Caffeine consumption was limited to no more than 4 servings per day and no caffeine within 6 h of bedtime, while vigorous physical activity was prohibited within 2 h of bedtime.

### 2.4. Measures

Sleep diary data were used to calculate daily total sleep time, sleep onset latency, wake after sleep onset, sleep efficiency, and daily sleep quality and morning drowsiness. Sleep diary items were based on the Consensus Sleep Diary [28]. Activity tracker data were used to calculate daily total sleep time, sleep efficiency, and percent of light, deep, and REM sleep, as these devices have demonstrated validity for estimating sleep and wake, and moderate accuracy for sleep staging, relative to polysomnography [29,30]. Then, daily values were averaged across weeks. The primary outcomes were sleep onset latency (measured by sleep diary) and percentage of REM sleep.

On Days 0, 7, and 30, participants completed an assessment battery. This battery included the Perceived Stress Scale (PSS) [31] to assess overall stress levels, the Profile of Mood States (POMS) [32] to assess current mood, the Insomnia Severity Index (ISI) [33] to assess overall insomnia symptom severity, the Center for Epidemiological Studies Depression Scale (CESD) [34] to assess depressive symptoms, and the Pittsburgh Sleep Quality Index (PSQI) [35] to assess overall sleep quality. Participants completed the JoggleResearch neurocognitive battery [36], which included the N-back test of working memory, a Visual Object Learning Task (VOLT) to assess visual learning and spatial working memory, a Motor Praxis Task (MPT) to assess sensory motor speed, an Abstract Matching task (AM), a Line Orientation Task (LOT), a Digital Symbol Substitution Task (DSST) for complex scanning and visual tracking, and a Balloon Analog Risk Task (BART) to assess risk-decision making, as well as a touchscreen Psychomotor Vigilance Task (PVT) [37] to quantify vigilant attention. At the end of the study (Day 30), unused investigational product was bought back to the investigators to determine subject adherence.

### 2.5. Statistical Analyses

All statistical analyses were conducted in R (v4.0.3, R Foundation for Statistical Computing, Vienna, Austria) using the ‘lme4’ and ‘lmerTest’ packages [38,39]. The primary outcomes were sleep onset latency measured by sleep diary and percentage of REM sleep measured by the activity tracker. Secondary outcomes included sleep diary total sleep time, wake after sleep onset, sleep efficiency, and sleep quality; activity tracker total sleep time, sleep efficiency, percentage of light, deep, and REM sleep; ISI, POMS, PSS, CESD, and PSQI scores; PVT attentional lapses and median reaction times; and neurocognitive functioning measured by the JoggleResearch battery. Pre–post comparisons were made using *t*-tests and chi-squared tests, while linear mixed-effects models assessed group, time, and group by time effects on study outcomes. Sleep diary and activity tracker outcomes were assessed 5 times (baseline, weeks 1–4), so there were enough degrees of freedom for models to include random intercepts and slopes. However, other outcomes were only assessed 3 times (baseline, day 7, day 30), and so those models only included random intercepts. All models were adjusted for sex and age, and significance was determined by Wald tests. Results are presented as mean (standard deviation) or N (percent) for summary statistics or beta coefficient (95% confidence interval) for regression models.

## 3. Results

### 3.1. Recruitment and Dropout

**Figure 1:**
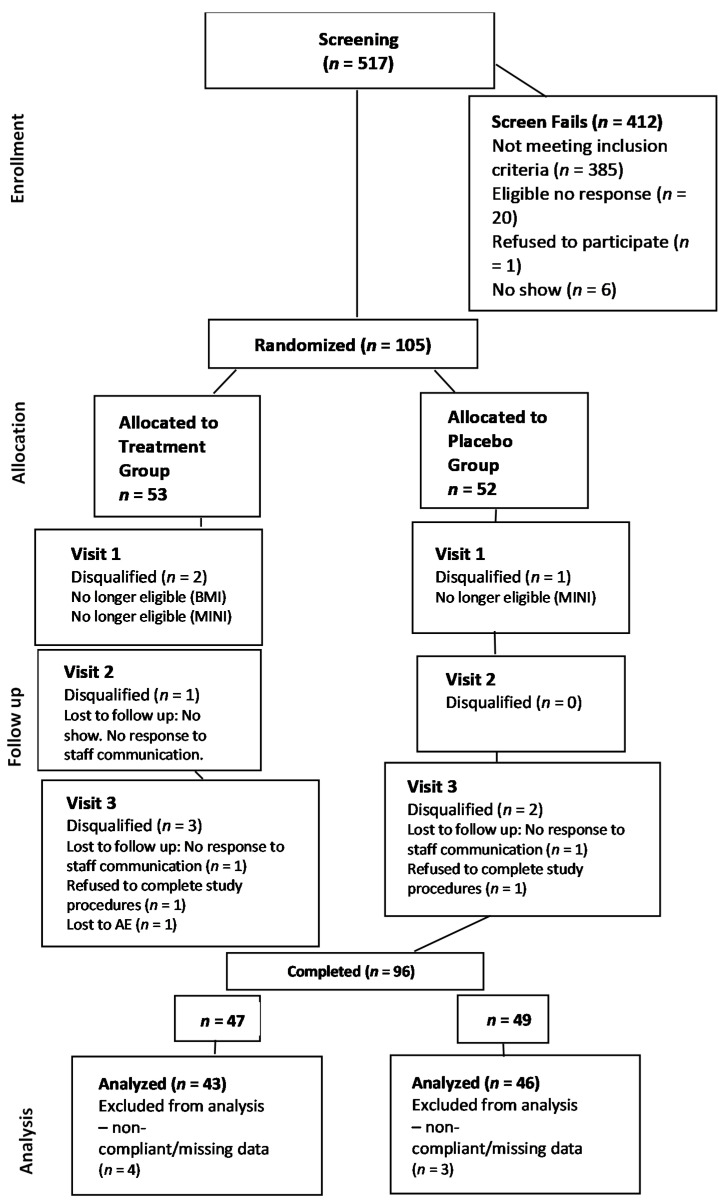
CONSORT flow diagram of study recruitment, randomization, and activities.

Recruitment and study completion are depicted in Figure 1. After screening 517 individuals, 105 subjects were randomized into the study. Of these, 96 subjects (91.4%) completed the study, although seven individuals (6.7%) were excluded due to data errors or adherence issues. This yielded a final sample of 89 participants (84.8%), with 43 receiving PBB and 46 receiving placebo. However, sleep data for one individual in the placebo group were unavailable due to a data error, so sleep outcomes were evaluated for N = 45 participants in the placebo group.

### 3.2. Sample Characteristics

The mean participant age was 31.5 (SD 8.1) years old, and the sample was 60% female. There were no differences between groups by age, sex, body mass index (BMI), or for any sleep diary variables. Activity tracker variables also showed no baseline group differences except for REM sleep, with the PBB group showing greater percentage REM than placebo (21.6 vs. 18.0, *p* = 0.022). These data are presented in Table 1. The subclinical sleep disturbances reported in the sample ranged across all elements of the PSQI except for use of a medication for sleep, which was minimal (Table S1).

### 3.3. Sleep Outcomes

**Figure 2:**
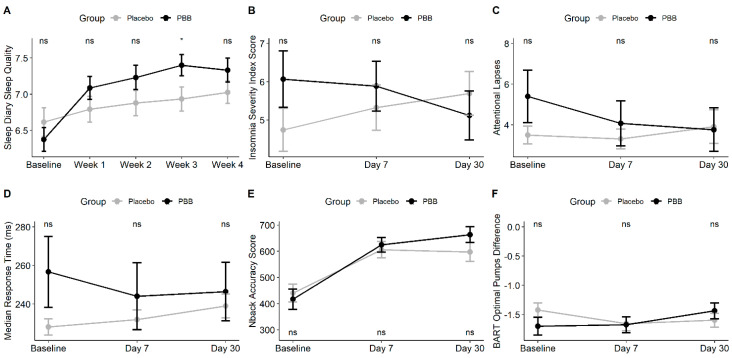
Mean (± SE) plots for outcomes with significant group by time effects, including sleep diary sleep quality (**A**), ISI score (**B**), lapses (**C**), PVT median reaction time (**D**), N-back accuracy (**E**), and BART optimal pumps difference (**F**). Tests of significance are between-group *t*-tests. * *p* < 0.05. ISI: Insomnia Severity Index, PBB: proprietary blend of botanical extracts, PVT: Psychomotor Vigilance Task, BART: Balloon Analogue Risk Task.

Pre–post comparisons and linear mixed-effects model results for the sleep outcomes are presented in Table 2. There were no pre–post differences or significant effects of time (*p* > 0.05) or group by time (*p* > 0.05) for sleep onset latency or percentage of REM sleep. However, there was a significant group by time effect for sleep quality (*p* = 0.008), where the PBB group showed a greater increase in sleep quality than placebo. This is shown in Figure 2A.

### 3.4. Questionnaire Outcomes

Pre–post comparisons and linear mixed-effects model results for questionnaire outcomes are presented in Table 3. There was a significant group by time effect for ISI score (*p* = 0.044), where the PBB group decreased significantly over placebo. These data are presented in Figure 2B.

### 3.5. Neurocognitive Outcomes

Pre–post comparisons showed that VOLT efficiency, N-back sensitivity, and N-back accuracy increased in the placebo group, while VOLT efficiency, N-back sensitivity, N-back accuracy, AM efficiency, and LOT efficiency increased in the PBB group. However, the only significant between-group differences were in AM efficiency. There were significant between-group effects for pre and post DSST efficiency, although this was because the PBB group scored consistently higher than the placebo group. In linear mixed models, the PBB group show no decline in neurocognitive outcomes when compared to placebo. Additionally, there were significant improvements in the PBB group for PVT attentional lapses (*p* = 0.035), and PVT median reaction time (*p* = 0.009), N-back accuracy (*p* = 0.044), and BART Optimal Pumps Difference (*p* = 0.022). For reference, the BART Optimal Pumps Difference refers to the number of pumps that maximizes an individual’s reward during the task, and then measures the group differences from that optimum. Thus, the improvement in this measure refers to participants more accurately assessing the risk of adding another pump. These results are presented in Table 4 and in Figure 2C-F.

## 4. Discussion

In this 30-day randomized double-blind placebo-controlled trial, PBB supplementation had no significant effects on sleep onset latency or percentage of time spent in REM sleep. However, PBB significantly improved self-reported sleep quality, sustained attention, and insomnia symptoms. Furthermore, PBB did not adversely affect neurocognitive functioning, and it even improved some elements of working memory, vigilant attention, and risk assessment. These data provide promising evidence that polyphenol compounds may improve sleep in individuals with subclinical sleep disturbances.

In contradiction to the primary study hypotheses, the use of PBB did not improve sleep onset latency or increase the percentage of REM sleep over placebo. Although no significant differences were noted, sleep onset latency at baseline was only 14–15 min and tended to improve in both groups, suggesting a placebo effect. Similarly, the lack of change in REM sleep may be due to the shorter baseline REM sleep in the placebo group, which regressed to the mean by the trial’s end. Placebo effects are common in sleep trials, including subjective sleep onset latency [40,41,42]. Additionally, post-trial interviews revealed that participants in both groups appreciated the consistent feedback on their sleep and used the study as a vehicle for maintaining better sleep habits.

Despite the null findings for sleep onset latency and percent REM sleep, the use of PBB resulted in modestly improved sleep quality, sustained attention, and insomnia symptoms over placebo. While a 1-point improvement on the ISI over the course of a month does not appear dramatic, participants’ ISI scores were not very large to begin with (4.7 in placebo, 6.1 in PBB), and so, a 1-point reduction in the PBB group represents an improvement of roughly 15% over baseline. At a mechanistic level, the polyphenol RA is known to decrease anxiogenic behavior and possibly increase endogenous GABA [16,17,25,43,44,45,46,47], thus leading to improved sleep and reduced anxiety/insomnia. Similarly, EGCG is known to improve mood [48], possibly through GABA-mediated inhibition [23]. These improvements in sleep and mood may be responsible for the reduced attentional lapses and shorter response times observed on the psychomotor vigilance task, although changes in cholinergic activity may also have supported sustained attention.

Finally, PBB showed no adverse neurocognitive effects and may have improved measures of vigilant attention, working memory, and risk assessment. This is notable considering that the sample was young, healthy, and had no major sleep disorders, which excludes several potential sources of neurocognitive impairment. On a clinical level, improved attention and risk assessment could reduce risk of injury in everyday activities such as driving a car, although further research would be needed to substantiate this claim. However, the primary point in this study was that PBB did not compromise neurocognitive functioning, which contrasts with other supplements that may improve sleep quality and insomnia symptoms but leave users with a morning hangover and grogginess that can lead to drowsy driving and impaired job performance. Together, these improvements in sleep quality, insomnia symptoms, and neurocognitive functioning indicate that polyphenol compounds could be a promising over-the-counter sleep aid to help individuals with subclinical sleep disturbances.

This study has a number of strengths, including the rigorous study design and use of validated measures of sleep and neurocognitive performance. Potential limitations include the narrow scope of trial participants, who were generally young, healthy, and free of clinical sleep disorders or significant sleep disturbances. Another limitation is that models were not adjusted for baseline differences in race/ethnicity and REM sleep, although the statistical difference in the former case is minor, and in the latter was not relevant given the null finding. Additionally, the lack of polysomnography data, which is the gold standard for evaluating sleep architecture, severely limits inferences on the effects of PBB on REM sleep. A further limitation is that neurocognitive testing was not conducted at a consistent time of day across individuals, meaning the results of neurocognitive testing could have been influenced by time-of-day testing effects. Future studies of polyphenol compounds as sleep aids should include individuals with a broader range of sleep disturbances, particularly older individuals, and they should compare PBB with other over-the-counter agents, such as diphenhydramine, melatonin, or other herbal remedies.

## 5. Conclusions

Despite numerous validated treatments for clinical sleep disorders, there are few effective options for individuals with subclinical sleep disturbances. Based on emerging evidence that polyphenol compounds can improve sleep, this randomized placebo-controlled trial evaluated the effects of a polyphenol blend on sleep, mood, and neurocognitive functioning. After 30 days, supplementation with a polyphenol blend improved sleep quality, reduced insomnia symptoms, and even improved some elements of neurocognitive functioning over placebo. These results indicate that polyphenols may have a role in over-the-counter treatment of subclinical sleep disturbances.

## Figures and Tables

**Table 1:** Baseline characteristics for the sample.

**Characteristic**	**Placebo**	**PBB ^1^**	***p*-value**
*N*	46	43	
Age	32.85 (8.62)	29.91 (7.17)	0.085
Sex			>0.9
Male	18 (40%)	17 (40%)	
Female	28 (60%)	26 (60%)	
Race/Ethnicity			0.048
White	25 (54.4%)	30 (69.8%)	
Black	3 (6.5%)	2 (4.7%)	
Hispanic	10 (21.7%)	10 (23.3%)	
Asian	8 (17.4%)	0 (0%)	
Native American	0 (0%)	1 (2.3%)	
BMI	24.4 (4.9)	23.9 (3.5)	0.6
**Sleep Diary**	**Placebo**	**PBB**	***p*-value**
Sleep Onset Latency (min)	14.02 (13.13)	14.94 (10.24)	0.7
Total Sleep Time (min)	406.08 (71.45)	408.33 (72.00)	0.9
Wake After Sleep Onset (min)	21.56 (19.88)	20.14 (18.28)	0.7
Sleep Efficiency (%)	83.09 (11.55)	83.57 (9.24)	0.8
Sleep Quality	6.62 (1.27)	6.37 (1.05)	0.3
Morning Refreshed	5.62 (2.01)	5.43 (2.02)	0.7
Morning Sleepiness	5.32 (1.79)	5.57 (1.84)	0.5
**Activity Tracker**	**Placebo**	**PBB**	***p*-value**
Total Sleep Time (min)	398.07 (70.65)	395.59 (67.38)	0.9
Light Sleep (%)	53.82 (16.49)	56.09 (9.01)	0.5
Deep Sleep (%)	15.68 (8.75)	17.30 (4.21)	0.3
REM Sleep (%)	18.03 (6.81)	21.60 (6.43)	0.022
Sleep Efficiency (%)	88.52 (2.66)	88.52 (2.10)	>0.9

^1^ PBB: Polyphenol Botanical Blend; Data are presented as Mean (SD) or *N* (%); Statistical tests performed: *t*-test; chi-square test of independence.

**Table 2:** Sleep diary and activity tracker outcomes.

Outcome	Placebo (*n* = 45)		PBB (*n* = 43)		Linear Mixed-Effect Modeling †	
**Sleep Diary**	**Pre**	**Post**	**Pre**	**Post**	**Time**	**Group by Time**
Sleep Onset Latency (min)	14.02 (13.13)	11.73 (10.23)	14.94 (10.24)	12.66 (7.62)	−0.53 (−1.3, 0.19)	0.01 (−1.0, 1.0)
Total Sleep Time (min)	406.08 (71.45)	425.36 (52.09)	408.33 (72.00)	428.54 (58.48)	6.0 (2.0, 10) **	−0.41 (−6.2, 5.3)
Wake After Sleep Onset (min)	21.56 (19.88)	18.03 (18.19)	20.14 (18.28)	16.15 (18.59)	−1.0, (−2.1, 0.08)	0.06 (−1.5, 1.6)
Sleep Efficiency (%)	83.09 (11.55)	87.71 (6.36)	83.57 (9.24)	86.42 (8.31)	1.2 (0.52, 1.9) ***	−0.49 (−1.5, 0.48)
Sleep Quality	6.62 (1.27)	7.02 (1.03)	6.37 (1.05)	7.33 (1.09) ***	0.10 (0.02, 0.17) *	0.11 (0.01, 0.22) *
Morning Refreshed	5.62 (2.01)	6.51 (1.56) *	5.43 (2.02)	6.60 (1.63) **	0.18 (0.06, 0.30) **	0.05 (−0.12, 0.23)
Morning Sleepiness	5.32 (1.79)	4.74 (1.39) *	5.57 (1.84)	4.56 (1.68) **	−0.13 (−0.24, −0.02) *	−0.06 (−0.22, 0.09)
**Actigraphy**	**Pre**	**Post**	**Pre**	**Post**	**Time**	**Group by Time**
REM Sleep (%)	18.03 (6.81)	19.67 (5.79)	21.60 (6.43)	21.61 (5.10)	0.26 (−0.16, 0.67)	−0.06 (−0.66, 0.54)
Total Sleep Time (min)	398.07 (70.65)	408.96 (48.17)	395.59 (67.38)	425.53 (40.97) *	4.3 (−0.35, 8.9)	5.2 (−1.4, 12)
Light Sleep (%)	53.82 (16.49)	53.46 (11.50)	56.09 (9.01)	56.26 (8.06)	−0.32 (−2.0, 1.3)	0.75 (−1.7, 3.2)
Deep Sleep (%)	15.68 (8.75)	15.57 (4.06)	17.30 (4.21)	16.37 (3.42)	−0.12 (−0.59, 0.35)	0.10 (−0.59, 0.78)
Sleep Efficiency (%)	88.52 (2.66)	88.32 (1.79)	88.52 (2.10)	88.29 (1.48)	0.00 (−0.16, 0.16)	−0.03 (−0.26, 0.20)

* *p* < 0.05 ** *p* < 0.01, *** *p* < 0.001. Pre–post data are compared within-groups. † Models adjusted for sex and age with random slopes and intercepts. Pre–post data are presented as mean (SD); regression outcomes are presented as β coefficients (95% CI).

**Table 3:** Pre–post changes and linear mixed-effects model results for questionnaire outcomes.

Treatment Group:	Placebo (*n* = 46)		PBB (*n* = 43)		Linear Mixed-Effect Models †	
**Questionnaires**	**Pre**	**Post**	**Pre**	**Post**	**Time**	**Group by Time**
Pittsburgh Sleep Quality Index	5.20 (2.41)	5.30 (2.11)	5.44 (2.91)	5.21 (2.24)	0.03 (−0.43, 0.50)	−0.15 (−0.82, 0.52)
Center for Epidemiological Studies Depression Scale	3.91 (3.43)	4.54 (4.55)	5.26 (4.62)	5.77 (5.59)	0.36 (−0.42, 1.1)	0.10 (−1.2, 1.0)
Perceived Stress Scale	12.96 (6.25)	14.35 (5.73)	14.91 (5.44)	15.28 (6.29)	0.70 (−0.17, 1.6)	−0.51 (−1.8, 0.73)
Insomnia Severity Index	4.74 (3.81)	5.70 (3.88)	6.07 (4.85)	5.12 (4.18)	0.48 (−0.14, 1.1)	−1.0 (−2.0, −0.03) *
Profile of Mood States						
Total Mood Disturbance	−3.54 (11.50)	0.33 (13.27)	0.49 (12.79)	1.05 (15.46)	2.0 (−0.33, 3.8)	−1.6 (−4.9, 1.7)
Tension	2.76 (2.18)	4.30 (3.75) *	4.09 (2.91)	4.05 (3.10)	0.79 (0.27, 1.3) **	−0.78 (−1.6, 0.01)
Depression	1.11 (1.90)	1.85 (3.75)	2.12 (3.88)	2.43 (3.83)	0.38 (−0.23, 1.0)	−0.22 (−1.1, 0.66)
Fatigue	3.78 (3.64)	4.26 (3.54)	4.47 (4.71)	4.52 (4.19)	0.23 (−0.45, 0.91)	−0.20 (−1.2, 0.78)
Anger	0.80 (1.61)	1.04 (1.69)	0.95 (2.06)	2.12 (3.49)	0.12 (−0.29, 0.53)	0.48 (−0.11, 1.1)
Confusion	4.30 (2.07)	4.63 (2.12)	4.42 (2.12)	4.64 (1.96)	0.17 (−0.20, 0.53)	−0.04, (−0.56, 0.49)
Vigor	16.30 (5.66)	15.76 (5.68)	15.56 (5.33)	16.71 (6.47)	−0.27 (−1.2, 0.67)	0.84 (−0.51, 2.2)

* *p* < 0.05, ** *p* < 0.01. † Models adjusted for age and sex. Pre–post data are presented as mean (SD); regression outcomes are presented as β coefficients (95% CI).

**Table 4:** Pre–post comparisons within and between groups on neurocognitive outcomes.

	Placebo		PBB		Linear Mixed-Effects Models †	
**Neurocognitive Test Battery**	**Pre**	**Post**	**Pre**	**Post**	**Time**	**Group by Time**
MPT Score	990.48 (6.03)	991.33 (6.41)	989.93 (6.36)	991.36 (10.11)	0.34 (−1.4, 2.1)	0.32 (−2.2, 3.0)
VOLT Efficiency	587.93 (94.21)	671.63 (104.59)	567.74 (102.02)	676.76 (89.48) ***	43 (30, 55) ***	11 (−6.2, 29)
N-Back Sensitivity (%)	56.09 (21.89)	67.53 (20.59)	50.55 (25.83)	71.91 (17.76) ***	5.6 (2.4, 8.7) ***	5.2 (0, 10.5)
N-Back Specificity (%)	87.47 (14.73)	92.18 (8.85)	90.60 (16.39)	94.47 (5.60)	2.4 (0.41, 4.4) *	−0.48 (−3.4, 2.4)
N-Back Accuracy	440.57 (228.62)	597.22 (246.46)	416.84 (251.24)	663.81 (191.34) ***	77 (46, 108) ***	48 (4.1, 92) *
AM Efficiency	535.41 (102.54)	578.61 (100.63)	539.35 (98.52)	617.19 (96.48) ***	21 (6.9, 35) **	17 (−3.0, 37)
LOT Mean Rotation Error (%)	48.33 (19.52)	48.93 (20.83)	55.12 (22.41)	46.71 (20.68)	0.69 (−2.1, 3.5)	−4.3 (−8.7, 0.15)
LOT Efficiency	776.93 (117.37)	802.91 (125.64)	763.65 (100.40)	820.71 (79.08) **	12 (−1.1, 26)	17 (−2.2, 36)
DSST Efficiency	940.48 (81.53)	954.24 (61.30)	962.70 (87.40)	980.00 (18.45)	6.9 (−2.0, 16)	1.6 (−11, 14)
BART Optimal Pumps Difference	−1.42 (0.82)	−1.60 (0.80)	−1.70 (0.99)	−1.44 (0.89)	−0.08 (−0.19, 0.02)	0.22 (0.06, 0.37) *
BART Accuracy	873.43 (147.84)	861.52 (141.57)	810.81 (181.53)	847.55 (149.21)	−5.4 (−29, 18)	25 (−9.3, 59)
**Psychomotor Vigilance Test**	**Pre**	**Post**	**Pre**	**Post**	**Time**	**Group by Time**
Attentional Lapses	3.50 (2.99)	3.91 (5.60)	5.40 (8.45)	3.76 (6.93)	0.21 (−0.43, 0.85)	−1.0 (−2.0, −0.12) *
Median Response Time (ms)	228.07 (28.45)	238.95 (42.22)	256.67 (120.57)	246.44 (97.79)	5.4 (0.33, 11) *	−11.0 (−18, −3.5) **
Mean Response Time (ms)	264.11 (48.17)	262.10 (56.24)	307.55 (208.07)	265.81 (99.30)	−1.0 (−17, 15)	−20.0 (−43, 2.4)

* *p* < 0.05, ** *p* < 0.01, *** *p* < 0.001. Pre–post data are compared within-groups. † Models adjusted for sex and age with random intercepts. Pre–post data are presented as mean (SD), while regression outcomes are presented as β-coefficients (95% CI).

## Data Availability

The data presented in this study may be available upon request from the corresponding author but are not publicly available due to non-disclosure agreements with the study sponsor.

## Supplementary Materials

The following are available online at https://www.mdpi.com/1660-4601/18/6/3044/s1, Table S1: Pre-post comparisons of sleep disturbances measured by the PSQI.
