# Supplementary material for: A Randomized, Double-Blind, Placebo-Controlled Trial of a Polyphenol Botanical Blend on Sleep and Daytime Functioning
Source: Int J Environ Res Public Health. 2021 Mar 16;18(6):3044. doi: 10.3390/ijerph18063044 (PMC8000032; doi:10.3390/ijerph18063044)
Supplement: Supplementary file 1 [file ijerph-18-03044-s001.pdf]

Table S1: Pre-post comparisons of sleep disturbances measured by the PSQI.						
	**Placebo**			**PBB**		
**PSQI Component Scores**	**Pre**	**Post**	**p-value**	**Pre**	**Post**	**p-value**
Sleep Quality			0.029			>0.9
0	13 (28%)	4 (8.7%)		6 (14%)	7 (17%)	
1	27 (59%)	38 (83%)		28 (65%)	28 (67%)	
2	6 (13%)	4 (8.7%)		8 (19%)	7 (17%)	
3	0 (0%)	0 (0%)		1 (2.3%)	0 (0%)	
Sleep Latency			0.15			0.061
0	17 (37%)	21 (46%)		14 (33%)	16 (38%)	
1	17 (37%)	21 (46%)		15 (35%)	21 (50%)	
2	11 (24%)	4 (8.7%)		9 (21%)	5 (12%)	
3	1 (2.2%)	0 (0%)		5 (12%)	0 (0%)	
Sleep Duration			0.14			0.061
0	8 (17%)	3 (6.5%)		11 (26%)	3 (7.1%)	
1	15 (33%)	12 (26%)		20 (47%)	21 (50%)	
2	22 (48%)	31 (67%)		10 (23%)	17 (40%)	
3	1 (2.2%)	0 (0%)		2 (4.7%)	1 (2.4%)	
Sleep Efficiency			0.5			0.6
0	34 (74%)	29 (63%)		26 (60%)	20 (48%)	
1	7 (15%)	13 (28%)		12 (28%)	14 (33%)	
2	3 (6.5%)	3 (6.5%)		2 (4.7%)	2 (4.8%)	
3	2 (4.3%)	1 (2.2%)		3 (7.0%)	6 (14%)	
Sleep Disturbances			0.2			0.4
0	5 (11%)	11 (24%)		4 (9.3%)	7 (17%)	
1	40 (87%)	35 (76%)		37 (86%)	31 (74%)	
2	1 (2.2%)	0 (0%)		2 (4.7%)	4 (9.5%)	
Medication			0.7			>0.9
0	43 (93%)	45 (98%)		42 (98%)	41 (98%)	
1	1 (2.2%)	1 (2.2%)		1 (2.3%)	1 (2.4%)	
2	1 (2.2%)	0 (0%)		0 (0%)	0 (0%)	
3	1 (2.2%)	0 (0%)		0 (0%)	0 (0%)	
Daytime Dysfunction			0.3			>0.9
0	18 (39%)	24 (52%)		20 (47%)	19 (45%)	
1	27 (59%)	22 (48%)		20 (47%)	20 (48%)	
2	1 (2.2%)	0 (0%)		3 (7.0%)	3 (7.1%)	
